# Sudden Cardiac Death Prediction in Non-ischemic Dilated Cardiomyopathy: a Multiparametric and Dynamic Approach

**DOI:** 10.1007/s11886-020-01343-9

**Published:** 2020-07-09

**Authors:** Daniel J. Hammersley, Brian P. Halliday

**Affiliations:** 1 Cardiovascular Research Centre, Royal Brompton Hospital, Sydney Street, London, SW3 6NP UK; 2 National Heart & Lung Institute, Imperial College London, London, UK

**Keywords:** Dilated cardiomyopathy, Sudden cardiac death

## Abstract

**Purpose of Review:**

Sudden cardiac death is recognised as a devastating consequence of non-ischaemic dilated cardiomyopathy. Although implantable cardiac defibrillators offer protection against some forms of sudden death, the identification of patients in this population most likely to benefit from this therapy remains challenging and controversial. In this review, we evaluate current guidelines and explore established and novel predictors of sudden cardiac death in patients with non-ischaemic dilated cardiomyopathy.

**Recent Findings:**

Current international guidelines for primary prevention implantable defibrillator therapy do not result in improved longevity for many patients with non-ischemic cardiomyopathy and severe left ventricular dysfunction. More precise methods for identifying higher-risk patients that derive true prognostic benefit from this therapy are required.

**Summary:**

Dynamic and multi-parametric characterization of myocardial, electrical, serological and genetic substrate offers novel strategies for predicting major arrhythmic risk. Balancing the risk of non-sudden death offers an opportunity to personalize therapy and avoid unnecessary device implantation for those less likely to derive benefit.

## Introduction

Non-ischaemic dilated cardiomyopathy (DCM) is a primary disease of the heart muscle characterized by left ventricular dilatation with systolic impairment in the absence of obstructive coronary artery disease or adverse loading conditions [1]. Although some studies estimate a prevalence of up to one in 250 [2], accurate contemporaneous estimates of this are not available. Multi-centre randomized controlled heart failure (HF) trials and large registry data typically report a non-ischaemic aetiology in 30–50% of patients with systolic HF, of which DCM is a leading cause [3, 4]. Although progressive refinement in therapy over the past three decades has resulted in improved survival, DCM remains a significant cause of mortality globally, principally driven by pump failure and sudden cardiac death (SCD) [5, 6].

Implantable cardiac defibrillators (ICDs) can recognise and promptly treat life-threatening arrhythmia, thereby offering protection against SCD. However, the selection of patients that will truly derive prognostic benefit from ICD therapy is a complex decision requiring balanced consideration of both individual arrhythmic risk and the competing risk of death from an alternate non-sudden cause. Current international guidelines informing such decisions remain rudimentary and their refinement represents a major unmet clinical need. In this review article, we evaluate current guidelines on SCD prevention in patients with DCM and additionally explore a number of parameters that may enhance risk stratification in this population.

## Current Guidelines and Landmark Studies

ICD implantation is recommended as a secondary prevention measure for patients with DCM who have survived an episode of ventricular arrhythmia with haemodynamic collapse where life expectancy exceeds 1 year [7, 8]. This indication remains relatively free of controversy due to a consistent evidence base which demonstrates reduction in both all-cause mortality (hazard ratio [HR], 0.72; 95% confidence interval [CI], 0.60–0.87; *P* = 0.0006) and SCD (HR 0.50; 95% CI 0.37–0.67; *P* < 0.0001) from ICD therapy compared with amiodarone on meta-analysis of three randomized controlled trials (RCTs) [9].

RCTs evaluating the utility of ICDs for primary prevention of SCD in DCM are notable for greater heterogeneity in their major findings (Table 1) [10]. The Sudden Cardiac Death in Heart Failure Trial (SCD-HeFT) enrolled patients with both ischaemic cardiomyopathy and DCM, LVEF < 35% and NYHA class II or III symptoms, demonstrating an all-cause mortality benefit across both aetiologies in patients randomized to single-chamber ICD implantation compared with placebo (HR 0.77; 97.5% CI 0.62–0.96; *P* = 0.007) [11]. The DEFINITE (Defibrillators in Non-Ischemic Cardiomyopathy Treatment Evaluation) trial enrolled patients with DCM with LVEF < 36%, NYHA class I–III symptoms and non-sustained ventricular tachycardia (NSVT) or frequent premature ventricular complexes and randomized to ICD versus optimal medical therapy (OMT). Conversely, no significant reduction in all-cause mortality was observed in the ICD group in DEFINITE compared to OMT (HR 0.65; 95% CI 0.40–1.06; *P* = 0.08) in spite of a significant reduction in SCD (HR 0.20; 95% CI 0.06–0.71; *P* = 0.006) [12]. Two earlier small RCTs, CAT and AMIOVIRT, were underpowered and consequently provided inconclusive results [13, 14]. ICD implantation for primary prevention of SCD is currently recommended for patients with DCM who have a left ventricular ejection fraction (LVEF) ≤ 35%, New York Health Association (NYHA) class II or III symptoms and treated with optimal therapy for at least 3 months with a life expectancy of > 1 year [7, 15]. This treatment paradigm is largely based on meta-analysis of above RCTs, which demonstrates an overall mortality benefit from ICD therapy (HR 0.74; *P* = 0.02) [16]. These results raised the possibility that previous trials may have been underpowered.

**Table 1:** Randomized trials of implantable cardioverter defibrillators

Study	*N*	Inclusion criteria	Intervention	Follow-up (median)	All-cause mortality	SCD
CAT [13]	104	LVEF < 30% NYHA 2–3	ICD vs OMT	23 months	Terminated early	
AMIOVIRT [12]	103	LVEF ≤ 35% NYHA 1–3 NSVT	ICD vs amio	24 months	Terminated early	
SCDHeFT (DCM cohort) [10]	1211	LVEF < 35% NYHA 2–3	ICD vs OMT vs amio	46 months	I 21.4%, C 27.9% (5 years) HR 0.73; 95% CI 0.50–1.07 *p* = 0.06	
DEFINITE [11]	458	LVEF < 36% NYHA 1–3 NSVT or PVCs	ICD vs OMT	29 months	I 12.2%, C 17.4% HR 0.65; 95% CI 0.40–1.06 *p* = 0.08	I 1.3%, C 6.1% HR 0.20; 95% CI 0.06–0.71 *P* = 0.006
DANISH [16]	1116	LVEF < 35% NYHA 2–3 (4 if CRT) NT-pro-BNP > 200 pg/ml	ICD vs OMT	68 months	I 21.6%, C 23.4% HR 0.87; 95% CI 0.68–1.12 *p* = 0.28	I 4.3%, C 8.2% HR 0.50; 95% CI 0.31–0.82 *p* = 0.005

Randomized trials investigating effect of implantable cardioverter defibrillators in patients with dilated cardiomyopathy without a history of haemodynamically unstable ventricular arrhythmia

*amio* amiodarone, *C* optimal medical therapy arm, *CI* confidence interval, *CRT* cardiac resynchronisation therapy, *HR* hazard ratio, *I* implantable cardioverter defibrillator therapy arm, *ICD* implantable cardioverter defibrillator, *LVEF* left ventricular ejection fraction, *NYHA* New York Heart Association, *NT-pro-BNP* N-terminal-pro-peptide brain natriuretic peptide, *NSVT* non-sustained ventricular tachycardia, *PVCs* premature ventricular complexes, *OMT* optimal medical therapy, *SCD* sudden cardiac death

(Reproduced with permission from: Halliday et al. Circulation [Internet]. 2017;136:215–31. Available from: http://circ.ahajournals.org/lookup/doi/10.1161/CIRCULATIONAHA.116.0271340) [9]

More recently, the DANISH (Defibrillator Implantation in Patients with Non-ischemic Systolic Heart Failure) study randomized symptomatic non-ischaemic heart failure patients with LVEF < 35% to ICD therapy versus optimal medical therapy. The results showed no all-cause mortality benefit in the ICD group versus control (HR 0.87; 95% CI 0.68–1.12; *P* = 0.28) in spite of a significant reduction in SCD in the ICD group (HR 0.50; 95% CI 0.31–0.82; *P* = 0.005). Notably, 58% of patients in each group received cardiac resynchronisation therapy (CRT) and the proportion of patients treated with guideline-based pharmacotherapy was higher than in earlier RCTs, reflecting contemporary clinical practice [17]. However, an updated meta-analysis integrating DANISH to prior RCTs has since demonstrated a mortality benefit from ICD therapy (odds ratio [OR] 0.77, 95% CI 0.64–0.93; *P* = 0.006) [18]. It is important to highlight that many patients from earlier trials included in this meta-analysis were not on contemporary heart failure therapies which reduce SCD. It is possible that modern therapy reduces the likelihood of gaining longevity from an ICD by reducing SCD.

The DANISH study illustrates several key concepts for SCD prediction and ICD therapy risk stratification in the current clinical landscape. Firstly, life-threatening arrhythmic events in this population are rare, as evidenced by the low rates of SCD in the control arm (46/560 [8%], over median 67.6 month follow-up) [17]. This finding is consistent with a larger analysis of 40,195 patients from 12 clinical trials investigating patients with systolic heart failure over a 19-year period, demonstrating a reduction in SCD by 44% over time due to the improved application of guideline-based therapy [19••]. Although not yet formally evaluated, novel therapies such as angiotensin receptor–neprolysin inhibitors (ARNIs) and sodium-glucose cotransporter 2 (SGLT2) inhibitors may further reduce this risk. In real terms, this means that most patients will have to survive many years to derive true prognostic benefit from an ICD. In light of this, it is not surprising that 85/131 (65%) of patients in the control arm of DANISH died due to causes other than SCD [20••], additionally illustrating the significance of the competing risk of death from alternate non-sudden cause in this population.

A further important consideration illustrated by DANISH relates to the dynamic evolution of risk with time and therapy. CRT is now a frequently utilized intervention for patients with non-ischaemic HF and in its own right promotes left ventricular reverse remodelling which reduces arrhythmic risk. It will also reduce SCD secondary to bradyarrhythmia. Subgroup analysis of the Multicentre Automatic Defibrillator Implantation Trial With Cardiac Resynchronization Therapy (MADIT-CRT) demonstrated marked reduction of appropriate ICD therapies in patients with LVEF normalization to a value > 50% (HR 0.24; 95% CI 0.07–0.82; *P* = 0.023) and reduction in those with modest LVEF improvement to a value of 36–50% (HR, 0.44; 95% CI 0.28–0.68; *P* < 0.001) [21]. These findings have been replicated by Manfredi et al. in a large single-centre retrospective analysis of patients undergoing CRT-defibrillator (CRT-D) implantation [22]. More recently, these findings have been reproduced on meta-analysis of six retrospective cohort studies (*n* = 1740) [23]. This concept is further illustrated by a subgroup analysis of the DEFINITE study evaluating patients with follow-up LVEF assessment in the absence of CRT. This demonstrated that 51% of patients underwent left ventricular reverse remodelling, defined as an improvement in LVEF by > 5%, and that this was associated with reduced mortality (HR 0.09; 95% CI 0.02–0.39; *P* = 0.001) [24]. No RCTs have demonstrated a benefit of CRT-D over CRT-P. CRT-P may be more cost-effective and at least as effective at reducing morbidity and improving mortality compared to CRT-D for patients who have a high chance of responding. The risk of SCD for such patients is low while the risk of inappropriate therapies remains high after improvement in LVEF [22, 23].

## Balancing the Risk of Sudden Death Versus Death from Competing Causes

It is clear that both the risk of SCD and the risk of death from competing non-sudden causes exist as independent and dynamic variables in patients with DCM. The current treatment paradigm utilizing a single measure of LVEF and NHYA class as a surrogate for evaluating the proportional risk of each of these variables is widely recognised as an inadequate determinant of ICD candidacy. While patients with lower LVEF are likely to have a higher absolute risk of SCD, their risk of death from heart failure is often proportionally higher, especially in patients with advanced age and/or comorbidity [25]. Consequently, ICD therapy will frequently not improve survival. Conversely, there exists a cohort of patients with a LVEF > 35% with lower absolute risk of SCD but a significantly lower competing risk of non-sudden death. In spite of the lower absolute risk of SCD, this cohort is more likely to survive longer and thus benefit from ICD therapy due to greater cumulative SCD risk exposure [26, 27]. Registry data identifies a significant proportion of SCD cases that occur in subjects with LVEF > 35% who would not fulfil current eligibility criteria for ICD implantation, illustrating the limited sensitivity of the current paradigm [28, 29]. Conversely, as a result of the low incidence of life-threatening arrhythmia with modern therapies and high competing risk of non-sudden death, many patients undergoing ICD implantation never receive therapy from their ICD in the form of anti-tachycardia pacing (ATP) or appropriate ICD discharge, illustrating the limited specificity of current practice [30].

Subgroup analysis of DANISH identified that younger patients with DCM may derive a mortality benefit from ICD implantation, whereas older patients do not [20••]. Post hoc analysis further characterized the association between ICD therapy and all-cause mortality, finding that this association decreased in a linear relationship with increasing age, suggesting that the age of 70 may represent the optimal cut off for recommending ICD therapy. A reduction in all-cause mortality was observed in patients with an ICD who were aged ≤ 70 (HR 0.70; 95% CI 0.51–0.96; *P* = 0.03) but not in patients aged > 70 (HR 1.05; 95% CI 0.68–1.62; *P* = 0.84) [31•].

Existing scoring systems offer the ability to explore the risk of death using a number of easily obtainable clinical, biochemical and therapy-based parameters. The Seattle Heart Failure model (SHFM) derives estimations of all-cause mortality which have been validated in large RCTs. This model has been extended to stratify by mode of death, confirming that as the severity of heart failure increases, there is a proportionally higher increase in the risk of HF death than SCD. This concept can be exemplified by considering a patient with a SHFM score of 4, which confers a sevenfold increase in risk of SCD and an 88-fold increase in risk of HF death compared to a patient with a SHFM score of 0 [25, 32]. Subsequently, the Seattle Proportional Risk Model (SPRM) was developed by modelling variables independently associated with a disproportionately higher risk of SCD, facilitating estimation of the proportional risk of SCD against non-sudden death [33]. A further post hoc study applying both SPRM and SHFM scoring to the DANISH cohort has now been reported, testing whether these models can enhance the precision of ICD selection. This demonstrates that ICD implantation is associated with a 37% relative reduction in all-cause mortality in patients with a SPRM score above the median and a 55% reduction in patients with both SPRM and SHFM scores above median, namely those patients with both a high proportional risk of SCD and a high overall mortality risk [34, 35].

**Fig. 1:**
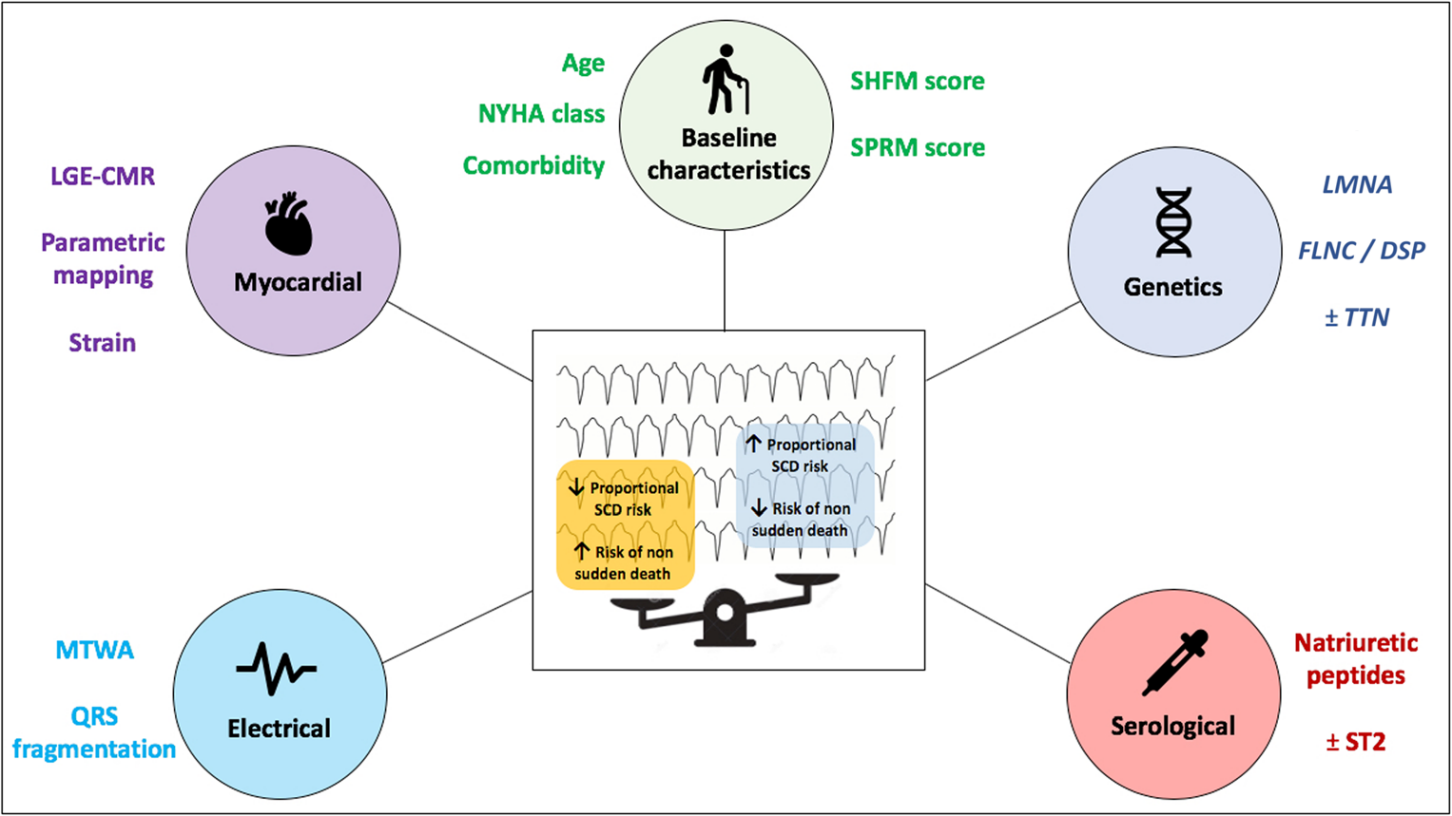
Contributory factors conferring risk of sudden or non-sudden death in patients with dilated cardiomyopathy. LGE-CMR, late gadolinium enhancement cardiovascular magnetic resonance; MTWA, microvolt T wave alternans; NHYA, New York Heart Association; SCD, sudden cardiac death; SHFM, Seattle Heart Failure Model; SPRM, Seattle Proportional Risk Model; ST2, suppression of tumorigenicity; *FLNC*, filamin C; *DSP*, desmoplakin; *TTN*, titin; *LMNA*, lamin AC

We take forward from these observations that the integration of simple clinical variables can improve estimation of proportional SCD risk against competing risk of non-sudden death. This can in turn improve our ability to identify patients with DCM that are most likely to benefit from primary prevention ICD therapy. Clinical variables should, however, be supplemented with additional parameters which can be broadly classified as relating to myocardial, electrical, serological and genetic markers of risk (Fig. 1).

## Myocardial Markers of Risk

### Late Gadolinium Enhancement (LGE-CMR)

Myocardial replacement fibrosis can be detected by late gadolinium enhancement cardiovascular magnetic resonance imaging (LGE-CMR). Replacement fibrosis is found in approximately 30% of patients with DCM, typically in the mid-wall of the septum [36]. The association between the presence of non-ischaemic LGE and SCD is well-established across numerous studies [37–41]. In a large prospective observational cohort study by Gulati et al., 472 patients with DCM were recruited and followed-up for a median of 64 months. A strong association was found between LGE presence on CMR and the composite arrhythmic endpoint (SCD or aborted SCD) (HR 4.61, 95% CI 2.75–7.74, *P* < 0.001) after adjustment for LVEF, with less powerful association demonstrated between with all-cause mortality (HR 2.43, 95% CI 1.5–3.92, *P* < 0.001) and a composite HF endpoint (HR 1.62; 95% CI 1.00–2.61; *P* = 0.049) [38]. The dose-response relationship between the LGE and risk of SCD has been evaluated subsequently in a larger cohort of patients with DCM; this relationship does not appear to be linear and the presence alone of LGE may be a better mark of risk than LGE extent [42]. The same study identified that the co-existence of septal and left ventricular free wall LGE was associated with an increased risk of SCD or aborted SCD [42]. A prospective study of patients with a less severe DCM phenotype and no pre-existing ICD indication illustrates that LGE remains a powerful predictor of SCD or aborted SCD in DCM in patients with LVEF > 40% (HR 9.2; 95% CI 3.9–21.8; *P* < 0.0001) [43•]. Importantly, these patients would not be eligible for a primary prevention ICD under current guidelines yet are likely to derive significant benefit due to a lower competing risk of non-sudden death. Computational modelling techniques have recently been used to further characterize LGE in patients with DCM, demonstrating extensive variation in fibrosis type and density and linking these to re-entry inducibility and arrhythmogenesis [44]. An alternative method evaluating LGE entropy, a measure of scar heterogeneity, was found to have significant independent predictive value for major arrhythmic events in a registry-based study of patients with DCM and primary prevention ICDs [45]. Overall, the additional added value of LGE assessment affords incremental prognostic value over echocardiography and thus is the gold standard imaging modality in the evaluation of patients with DCM [46].

### Cardiovascular Magnetic Resonance Parametric Mapping

T1 mapping is a non-invasive technique that can be used to measure diffuse interstitial fibrosis across a spectrum of cardiac conditions [47]. Puntmann et al. demonstrated an association between native T1 values and both all-cause mortality (HR 1.1; 95% CI 1.06–1.15; *P* < 0.001) and a composite HF endpoint (HR 1.1; 95% CI 1.05–1.1; *p* < 0.001 [48]. It has been proposed that a relationship between diffuse fibrosis and arrhythmogenesis may exist. Accordingly, T1 mapping was subsequently found to be predictive of major arrythmia in patients with both ischaemic cardiomyopathy and DCM prior to ICD implantation [49]. Further work to validate this technique is awaited. The true incremental value in addition to LGE remains unclear.

### Myocardial Strain

Myocardial strain measures the degree of myocardial deformation from a fixed point throughout the cardiac cycle and can be evaluated using either echocardiography or CMR [50]. Left ventricular (LV) global longitudinal strain (GLS) offers a reproducible alternative measure of LV contractile performance to LVEF and may be more sensitive to subtle dysfunction [51]. Myocardial strain has also been associated with survival across a number of cardiovascular condition [52–54]. Romano et al. have reported on the association between LV GLS and all-cause mortality in a large multi-centre cohort of patients with LVEF <50% (*n* = 1012), demonstrating that each 1% worsening in GLS was associated with an ﻿89.1% increased risk of death after adjustment for clinical and imaging variables including EF and LGE (HR 1.891 per %; *P* < 0.001) across patients with both ischaemic cardiomyopathy and DCM [55]. A single retrospective echocardiography-based study found no association between LV GLS and ventricular fibrillation or sustained ventricular tachycardia [56]. Whether myocardial strain can identify patients most likely to benefit from ICDs or whether it may be better at stratifying heart failure death rather than arrhythmic events is unclear.

### Future Horizons

Diffusion-tensor cardiovascular magnetic resonance (DT-CMR) is a novel CMR technique that facilitates non-invasive interrogation of the cardiac microstructure at the level of the cardiomyocyte. Existing studies have demonstrated microstructural abnormalities in patients with DCM [57, 58], however, the utility of DT-CMR to predict SCD has not been assessed. Of note, a single study of 50 patients with hypertrophic cardiomyopathy found that low fractional anisotropy was associated with ventricular arrhythmia (*P* = 0.007) [59]. Exploring whether this relationship can be reproduced in patients with DCM offers both a potential new imaging biomarker of arrhythmic risk and possible mechanistic insights into the microstructural pathophysiology of arrhythmogenesis.

There is growing interest in the interface between advanced cardiac imaging with artificial intelligence and machine learning [60]. Such approaches have already been developed and validated for CMR image segmentation, motion and deformation analysis. However, there is enormous potential from additionally using these techniques to extract huge datasets and model new markers of risk in patients with DCM. Studies evaluating the potential utility of this application are underway [61].

## Electrical Markers of Risk

Numerous studies have evaluated the association between established surface electrical or invasive electrophysiological investigations and major arrhythmic risk. However, a lack of consistent findings has hampered their integration into current guidelines.

### Microvolt T Wave Alternans

Microvolt T wave alternans (MTWA) is a measure of beat-to-beat variation in T wave amplitude. MTWA represents a marker of electrical instability and consequently may provide an opportunity to measure arrhythmic susceptibility. Meta-analysis of eight studies (*n* = 1456) evaluating patients with DCM found that an abnormal MTWA test conferred a major arrhythmic relative risk of 2.99 (95% CI 1.88–4.75) [62]. A further meta-analysis has suggested that this association may be most pronounced in patients taking beta-blockers [63].

### QRS Fragmentation

A lack of consistency exists between the major findings of studies exploring the association between QRS fragmentation and major arrhythmia. The largest prospective study of both ischaemic and non-ischaemic HF patients found no association between QRS fragmentation and arrhythmic mortality [64]. By contrast, Das et al. demonstrated strong association between QRS fragmentation and ventricular arrhythmia in patients with DCM (HR 15.09, 95% CI 3.30–69.06), and this association was further supported by subsequent meta-analysis (OR 6.73; 95% CI 3.85–11.76; *P* < 0.001) [65, 66].

### Heart Rhythm Monitoring

Historic studies investigating the association between non-sustained ventricular tachycardia (NSVT) and future major life-threatening arrhythmic events in patients with heart failure are notable for a lack of uniformity in their major conclusions, across both ischaemic and non-ischaemic aetiology [67, 68]. More recently, in a large registry of patients with DCM treated with OMT, no clear association was found between NSVT on Holter monitoring and major ventricular arrhythmic events [69]. Notably, this registry did observe that in patients with DCM and LVEF > 35%, the number and length of NSVT runs were associated with major ventricular events [69]. It is therefore possible that this may be most effective at risk-stratifying patients with less severe ventricular dysfunction.

### Ventricular Arrhythmia Inducibility

The inducibility of ventricular arrhythmia in the cardiac catheter lab has historically been recognised as an invasive method for evaluating ventricular arrythmia susceptibility in a broad range of cardiovascular conditions. The routine use of this technique has largely fallen out of favour in the wake of the MUSTT trial, which demonstrated poor negative predictive value [70]. In patients with DCM, a single prospective study found that inducible ventricular arrhythmia on programmed ventricular stimulation was not predictive of future major arrhythmic events [71].

### Autonomic Dysregulation

The autonomic nervous system is a key determinant of cardiac electrophysiology [72]. Consequently, autonomic dysfunction has been implicated in ventricular arrhythmogenesis, by generating myocardial heterogeneity in conduction and refractory periods [73]. Electrical parameters used as surrogates for autonomic dysfunction include heart rate variation and heart rate turbulence. Baroreflex sensitivity is a further measure of autonomic tone which integrates variation in both heart rate and blood pressure. Initial studies in patients with ischaemic heart disease indicated markers of autonomic function, particularly heart rate turbulence, predicted sudden cardiac death or aborted sudden cardiac death [74]. However, studies evaluating the association between markers of autonomic function and major arrhythmia in patients with DCM have failed to deliver consistent findings with no association demonstrated on meta-analysis [66, 75–77].

## Serological Markers of Risk

To date, there is no single peripheral circulatory biomarker that has been adopted as a recognised predictive biomarker of major arrhythmic episodes in patients with dilated cardiomyopathy. Higher levels of natriuretic peptides are, however, associated with increased all-cause mortality and major heart failure events in both DCM and ischaemic cardiomyopathy, reflecting their association with deterioration in cardiac function and heart failure progression [78]. Natriuretic peptides may therefore play a role in identifying those less likely to benefit from ICD therapy, namely those with a lower proportional risk of SCD and higher risk of HF death. This hypothesis was supported by a subgroup analysis of DANISH, in which patients with NT-pro BNP > 1177 p/mL derived no mortality benefit from ICD therapy compared to OMT (HR 0.99; 95% CI 0.73–1.36; *P* = 0.96). By contrast, patients with NT-pro BNP < 1177 p/mL did derive mortality benefit from ICD therapy (HR 0.59; 95% CI 0.38–0.91; *P* = 0.02) [17].

Suppression of tumorigenicity 2 (ST2) is a member of the interleukin-1 receptor family and is involved in signalling during adverse cardiac remodelling and fibrosis [79]. A single study of patients with coronary artery disease found that soluble ST2 and high-sensitivity troponin were associated with sudden cardiac death [80]. Two studies have found elevated soluble ST2 levels to be associated with sudden cardiac death in patients with both ischaemic and non-ischaemic heart failure [81, 82], however, the utility of soluble ST2 to improve SCD prediction in DCM remains unexplored.

In the absence of further studies demonstrating clear association between circulating biomarkers and major arrhythmia in patients with DCM, for now, their principle utility remains a measure of heart failure severity. Novel high-throughput experimental approaches for molecular phenotyping, including plasma proteomics and metabolomics, may afford the discovery of new biomarkers of risk and require further evaluation.

## Genetic Markers of Risk

The proportion of patients with familial DCM has previously been estimated at 20–30% [83]. Genetic characterization of a large outpatient cohort of patients with DCM (*n* = 1040) identified a genetic cause in 17% of the cohort, with 12 of the genes evaluated demonstrating robust disease association [84]. Unlike fibrosis on LGE-CMR or elevated natriuretic peptides observed in advanced disease, genetic variants represent a static variable present at disease outset. Stratifying risk based on genotype therefore offers an opportunity for early intervention in patients at very risk. However, in the vast majority of cases, DCM results from a complex interaction between genetic susceptibility and environmental triggers. Furthermore, many variants are associated with incomplete penetrance and variable expressivity. Thus, for most patients with DCM, genetic testing alone is an inadequate isolated modality to delineate risk and serves better as an adjunct complementing other multi-parametric measures. One notable exception to this rule is in patients with pathogenic mutations to *LMNA*, the gene that encodes the Lamin A and C proteins, which form components of the nuclear envelope. Laminopathy is associated with a particularly malignant disease characterized by high penetrance, early ventricular arrhythmia, atrioventricular (AV) block and progression to advanced heart failure. The high risk of ventricular arrhythmia, coupled with the risk of bradyarrhythmia from AV block, puts patients with disease-causing LMNA mutation at especially high risk of SCD and supports a significantly lower threshold for ICD implantation [85, 86].

The most common pathogenic variant associated with DCM are truncating variants of *TTN* (TTNtv), the gene encoding the giant sarcomeric protein titin. Studies evaluating arrhythmic risk in TTNtv DCM have suggested that these patients are susceptible to early atrial fibrillation and NSVT [87] and that those with an ICD are more likely to have appropriate therapies compared to DCM patients without TTNtv [88]. Conversely, other studies have demonstrated high rates of left ventricular reverse remodelling in patients with TTNtv [89] and a comparable incidence of adverse events to the greater population of patients with DCM [90], meaning that the true burden and significance of their arrhythmic risk remains unclear.

A further subgroup of patients exists with variable and overlapping features common to both dilated and arrhythmogenic cardiomyopathy phenotypes. Truncating mutations to *FLNC*, which encodes the filamin cytoskeletal protein, are one such group. The largest study evaluating patients with *FLNC* mutations and their families demonstrated high penetrance, high rates of sustained VT (18%) and frequent SCD (15%), with a proportionally lower risk of major heart failure events [91]. The authors recommend early ICD therapy in such patients, although consideration should be given to the potential impact of selection bias when interpreting the findings of this study. Mutations to the *DSP* gene, encoding desmoplakin, represent another condition co-existing between the arrhythmogenic and dilated cardiomyopathy spectrum, associated with high rates of ventricular arrhythmia and SCD with or without left ventricular dysfunction [92–94]. A study evaluating the association between genetic mutation status and arrhythmic risk in patients with DCM found that pathogenic desmosomal mutations conferred significantly higher major arrhythmic risk compared with variant-negative patients, independently of LVEF and akin to the risk of laminopathy [95•]. A recent expert consensus statement from the Heart Rhythm Society recognised the higher risk of sudden death associated with both *FLNC*- and *DSP*-associated cardiomyopathy [96].

DCM associated with muscular dystrophies represents another distinct group of patients. In patients with Duchenne muscular dystrophy, SCD generally occurs in patients with both respiratory and cardiac failure and it remains unclear whether these patients derive equivalent benefit from ICD therapy as other DCM subgroups, especially given their conflated risk of non-sudden death due to the multi-system nature of disease [97].

## Conclusions

SCD in patients with non-ischaemic dilated cardiomyopathy is an increasingly rare but devastating event. The variable nature of disease progression or regression and the significant competing risk of non-sudden death represent major challenges in delineating true major arrhythmic risk when evaluating ICD candidacy. The current guidelines for predicting SCD and guiding ICD implantation are well recognised as being inadequate. Adopting an approach that recognises the dynamic nature of disease and integrates a multi-parametric evaluation of myocardial, electrical, serological and genetic substrate offers promising potential for a more precise and personalised method for predicting SCD.
